# Environmental Conditions Influence Induction of Key *ABC-Transporter* Genes Affecting Glyphosate Resistance Mechanism in *Conyza canadensis*

**DOI:** 10.3390/ijms17040342

**Published:** 2016-04-20

**Authors:** Eleni Tani, Demosthenis Chachalis, Ilias S. Travlos, Dimitrios Bilalis

**Affiliations:** 1 Laboratory of Plant Breeding, Department of Crop Science, Agricultural University of Athens, Iera Odos 75, 11855 Athens, Greece; etani@aua.gr; 2 Laboratory of Weed Science, Benaki Phytopathological Institute, S. Delta 8, 14561 Athens, Greece; 3 Laboratory of Agronomy, Department of Crop Science, Agricultural University of Athens, Iera Odos 75, 11855 Athens, Greece; htravlos@yahoo.gr (I.S.T.); bilalis@aua.gr (D.B.)

**Keywords:** *Conyza canadensis*, glyphosate resistance, *ABC-transporter* genes, *EPSPS*, environmental factors

## Abstract

*Conyza canadensis* has been reported to be the most frequent weed species that evolved resistance to glyphosate in various parts of the world. The objective of the present study was to investigate the effect of environmental conditions (temperature and light) on the expression levels of the *EPSPS* gene and two major *ABC*-*transporter* genes (*M10* and *M11*) on glyphosate susceptible (GS) and glyphosate resistant (GR) horseweed populations, collected from several regions across Greece. Real-time PCR was conducted to determine the expression level of the aforementioned genes when glyphosate was applied at normal (1×; 533 g·a.e.·ha^−1^) and high rates (4×, 8×), measured at an early one day after treatment (DAT) and a later stage (four DAT) of expression. Plants were exposed to light or dark conditions, at three temperature regimes (8, 25, 35 °C). GR plants were made sensitive when exposed to 8 °C with light; those sensitized plants behaved biochemically (shikimate accumulation) and molecularly (expression of *EPSPS* and *ABC*-genes) like the GS plants. Results from the current study show the direct link between the environmental conditions and the induction level of the above key genes that likely affect the efficiency of the proposed mechanism of glyphosate resistance.

## 1. Introduction

Glyphosate (*N*-phosphonomethyl glycine) is the most widely used herbicide in the world due to its high efficiency, broad-spectrum capacity and systemic mode of action. It binds to the active site of 5-enolpyruvylshikimate 3-phosphate synthase (EPSPS), a key enzyme of the shikimate pathway, antagonizing its natural substrate phosphoenol pyruvate. Thus, it inhibits the synthesis of crucial amino acids and other compounds causing subsequent plant death [1,2,3].

The wide use of glyphosate-resistant (GR) crops has led to an enormous increase in the application of glyphosate, as it constitutes a low-cost and highly effective weed control technology. However, its repeated and intensive use has exerted a high selection pressure on weed populations and has led to the evolution of glyphosate resistance weeds. To date, 25 weed species have evolved resistance to glyphosate worldwide [4], jeopardizing the efficiency of weed management programs in modern agriculture [5]. Thus, understanding the mechanism of glyphosate resistance in weeds is a prerequisite to guarantee its continued use [6].

Horseweed (*Conyza canadensis* L.), which belongs to the Asteraceae family, was the first broadleaf weed to evolve glyphosate resistance [7]. Especially in Mediterranean regions including Greece, *Conyza* spp. is the most difficult to tackle weed in perennial crops. Prolonged and exclusive use of glyphosate, combined with the lack of integrated weed management approaches, have mainly contributed to the evolution of tolerant and/or resistant biotypes in many orchard regions containing among others olives, grapes and citrus [8,9,10].

According to Sammons and Gaines [11] the glyphosate resistance mechanisms reported so far include: (1) mutations at the target site of the key enzyme *EPSPS* [12,13]; (2) gene amplification of *EPSPS* [14]; (3) limited absorption and/or translocation of the herbicide [15,16]; (4) changes in the sequestration of glyphosate to vacuoles [17]; (5) faster metabolism of the herbicide in resistant plants [18]; (6) rapid mature leaf necrosis resulting in reduced translocation in ragweed (*Ambrosia trifida*) [19]; and (7) the recently proposed synchronization of the overexpression of *EPSPS* and *ABC-transporter* genes [20]. As indicated in several reports, resistant weeds could combine several glyphosate resistance mechanisms within populations and within individuals [11].

High light intensity and elevated temperatures ameliorate glyphosate performance by enhancing the rapid absorption by the plant, as well as its accumulation and translocation [21]. Regarding the influence of environmental conditions to glyphosate efficacy, as a general rule it can be pointed out that glyphosate is more effective under higher temperatures and ambient light conditions due to elevated levels of plant metabolism as a result of an increased vegetative growth [21,22,23]. On the contrary, decreased glyphosate absorption and translocation is manifested in sub-optimal environmental conditions, resulting in a lower glyphosate efficacy on treated plants. Earlier studies have addressed how environmental conditions might affect levels of glyphosate resistance in various other weeds. Researchers have shown that resistance to both glyphosate and paraquat (thought to be dependent on vacuolar sequestration) is diminished at low temperatures [24,25]. Moreover, the analysis of an *Arabidopsis* GR mutant that was dysfunctional in perceiving light, further supported previous observations that light quality and intensity differentiates herbicide efficacy [26].

Results from our previous study in *C. canadensis*, clearly revealed that the glyphosate resistance mechanism involves a synchronized induction of *EPSPS* and *ABC-transporter* genes [20], supporting the concept that glyphosate resistance mechanisms can be quite complex [11]. Former studies showed that subjecting GR-plants to low temperatures could make those plants sensitive to glyphosate due to its higher vacuolar sequestration [24]. However, there has been no report so far regarding the effects of environmental conditions on the expression levels of the aforementioned key genes. Therefore, our main objective was to elucidate how two environmental conditions (temperature, light) affect the induction of key genes such as *EPSPS* and *ABC-transporters* in glyphosate susceptible (GS) and GR plants aiming to further understand the mechanism of glyphosate resistance in weed species such as *C. canadensis*.

## 2. Results

### 2.1. Shikimate Measurements

**Figure 1:**
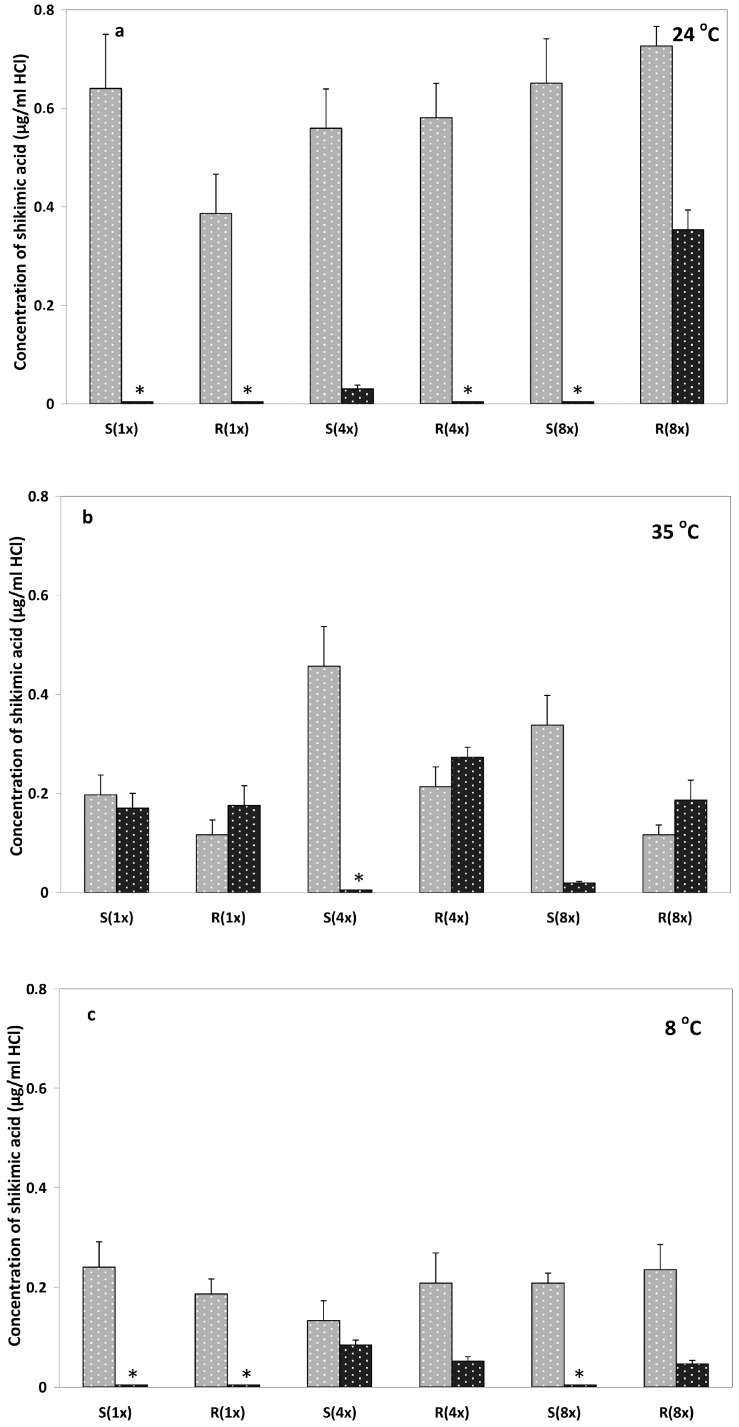
Shikimic acid determination at four days after treatment (DAT). Shikimate levels are reported as micrograms of 1 shikimic acid per mL of HCl solution (**a**) at 24 °C; (**b**) at 35 °C; (**c**) at 8 °C (grey columns represent measurements taken from plants grown in light conditions, whereas black columns in dark conditions). Asterisks denote values close to zero.

There was a clear differentiation for shikimate measurements between light and dark conditions, especially at 35 °C (Figure 1). Generally, in light conditions, shikimate levels were higher than in dark conditions. Moreover, in light conditions shikimate accumulated at lower concentrations in the resistant population compared to the susceptible one, especially at normal glyphosate doses.

At 24 °C, in light conditions results were similar to our previous work; nevertheless, the differences between the GR-population and the GS-population were smaller. In the dark, shikimate accumulation was very low, with the only exception of GR-population after high glyphosate load (0.35 μg/mL HCl) (Figure 1a).

At 35 °C, shikimate accumulation was lower in the GR-population compared to the GS- at all glyphosate rates in light conditions. However, in the dark results were reversed (Figure 1b).

Finally, at 8 °C the levels of shikimate were very low, especially in dark conditions (black columns), with no significant differences between the GR- and the GS-populations (Figure 1c).

### 2.2. Expression Analysis of ABC-Transporter-Like Genes and EPSPS Gene

In our previous work, we studied the expression levels of 5 *ABC-transporter* genes and the *EPSPS* gene [20]. Two *ABC-transporter* genes, namely *P3* and *M7*, showed a small increase in their relative expression at both glyphosate doses, thus they were not studied in the present work. Below, the results of our experiments in the three temperature regimes are presented.

#### 2.2.1. Relative Expression of *M10*, *M11* and *EPSPS* Genes at 24 °C

The results are in agreement with our previous results for 24 °C with small changes in the magnitude of induction for each gene in the resistant population [20].

**Figure 2:**
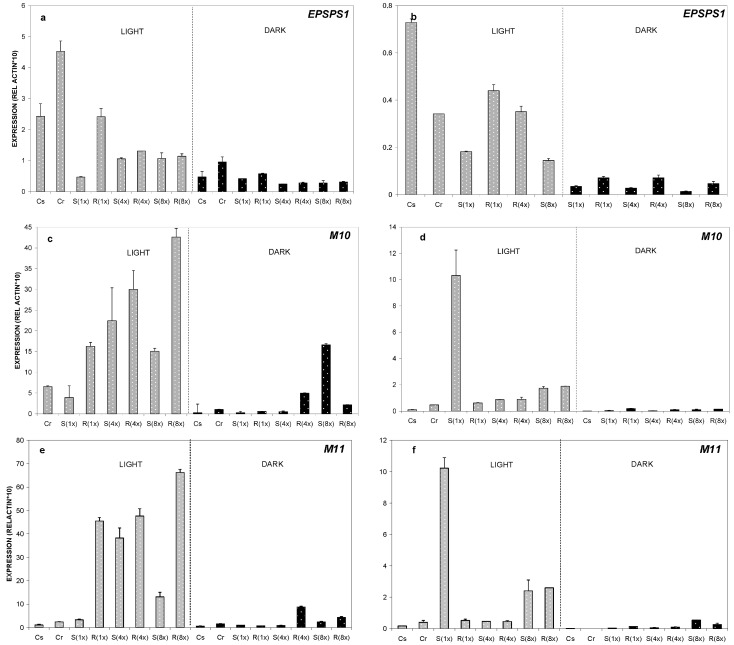
Relative expression of *EPSPS1* (**a**,**b**), *M10* (**c**,**d**), and *M11* (**e**,**f**) genes analyzed by real time PCR. Leaf samples were taken from plants grown at 24 °C. Relative quantification was obtained through the delta-delta-*C*t algorithm (2^ΔΔ*C*t^) method using *actin* as the reference gene. As a reference sample, the untreated (control) plants from the susceptible biotype (Cs) was chosen. Data represent the average from three biological replicates and the error bars indicate the standard deviation. The following plant–treatment combinations were studied: Cs susceptible biotype-untreated control; Cr resistant biotype-untreated control; S(1x) susceptible biotype- glyphosate sprayed, dose 1x; R(1x) resistant biotype-glyphosate sprayed, dose1x; S(8x) susceptible biotype-glyphosate sprayed, dose 8x; R(8x) resistant biotype-glyphosate sprayed, dose 8x. Relative expression ratios were estimated one and four days after glyphosate treatment (1 DAT—diagrams on the left and 4 DAT—diagrams on the right).

Concerning the *EPSPS* gene, at one day after treatment (DAT) statistically significant differences were observed between GR- and GS-populations at the normal glyphosate load (six-fold up regulation compared to the GS-population) (Figure 2a). In the dark, for all treatments, the expression ratio was very low. However, at four DAT there was no significant variation in *EPSPS* relative expression ratio between the GR- and the GS-population (Figure 2b).

Regarding *ABC-transporters*, *M10* displayed a statistically significant augmentation of relative expression at the high glyphosate application rate (three-fold increase) in the GR-population compared to the GS-population (Figure 2c). At four DAT, an increase of *M10* relative expression ratio was observed in the GS population. Induction of the *M10* gene was eliminated under dark conditions with the exception of GS at high glyphosate dose (Figure 2d).

For the *M11* gene at one DAT, statistically significant differences occurred at both glyphosate doses, between GR- and GS-populations (Figure 2e). Under dark conditions at the 4X dose, a marked increase in expression (seven-fold) was evidenced for *M11* in the GR-population. At four DAT results were almost identical to *M10* expression for both populations (Figure 2f).

#### 2.2.2. Relative Expression of *M10*, *M11* and *EPSPS* Genes at 35 °C

**Figure 3:**
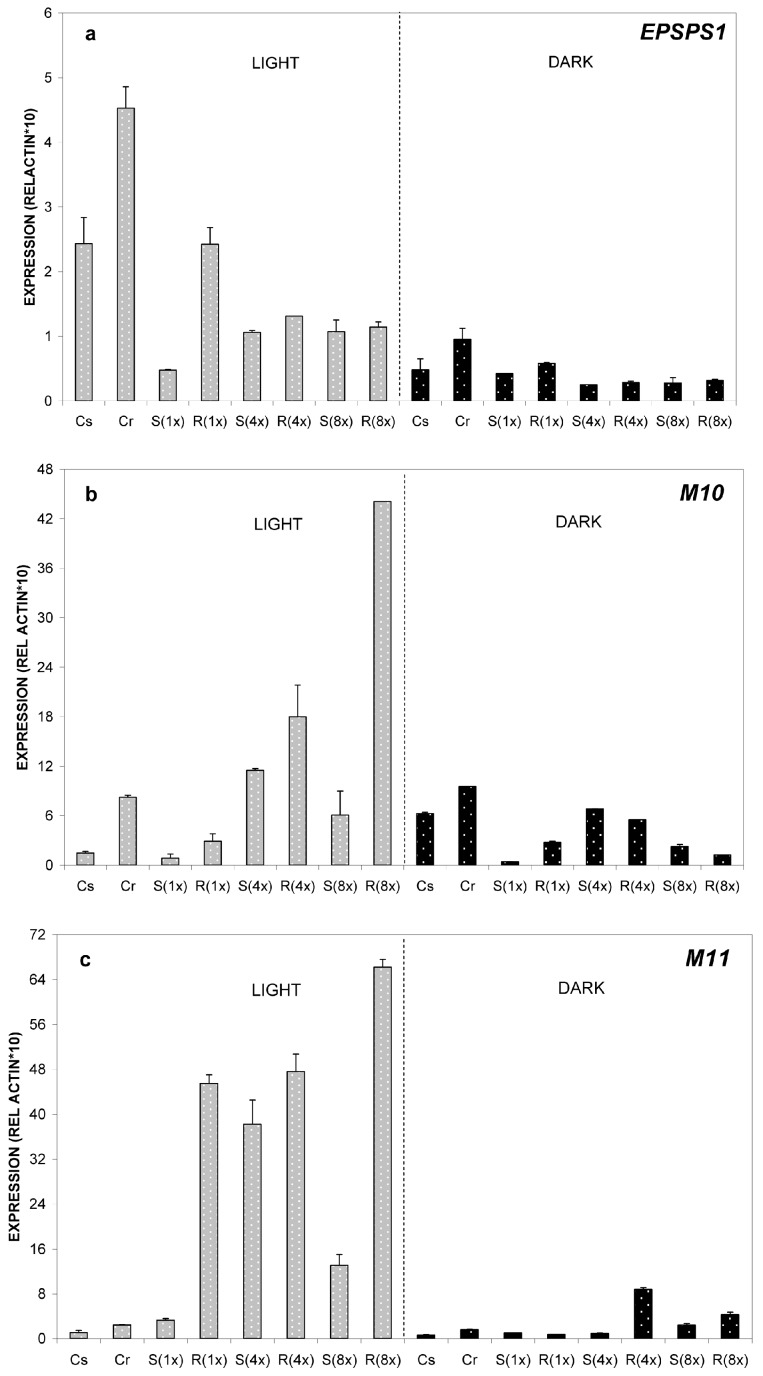
Relative expression profiles of *EPSPS1* gene (**a**) and of the *ABC*-*transporter* genes *Μ10* (**b**) and *M11* (**c**). Samples were taken from plants grown at 35 °C. Relative quantification was obtained through the 2^ΔΔ*C*t^ method using actin as the reference gene. Leaves from the untreated (control) plants of the susceptible biotype (Cs) were used as the reference sample. Explanation of samples abbreviations was given on Figure legend 2. Relative expression ratios were estimated after 1 DAT. Data represent the average from three biological replicates and the error bars indicate the standard deviation.

Regarding *EPSPS* relative expression, the differences that were detected between GS- and GR-populations were not statistically significant. In dark conditions, differences in the relative expression between the two populations were even smaller (Figure 3a).

Results were very similar for *M10* and *M11* genes to those obtained at 24 °C. Interestingly, the most profound differences of relative expression ratio for both *ABC-transporter* genes between GS and GR were observed at this temperature. More specifically, there was detected a 7.3-fold induction of the *M10* gene at high glyphosate load in the GR-population compared to the GS-population and a 15.8-fold difference in expression ratio of *M11* gene between the two populations (Figure 3b,c). Notably under dark conditions, the expression ratio of *M11* was high for both populations, especially for the GR one.

Finally, no detection of any of the genes was possible at four DAT at both light/dark conditions.

#### 2.2.3. Relative Expression of *M10*, *M11* and *EPSPS* Genes at 8 °C

**Figure 4:**
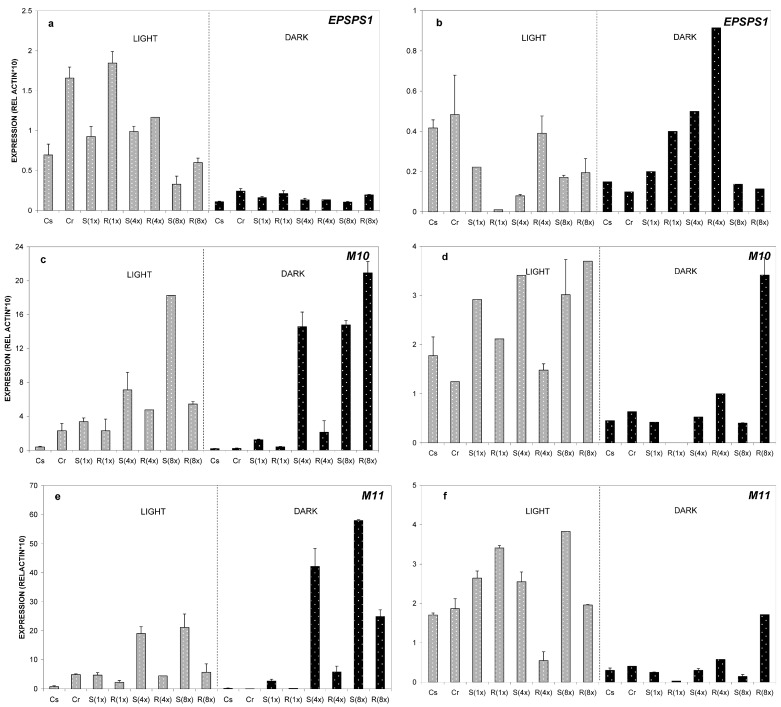
Relative expression of *EPSPS1* (**a**,**b**), *M10* (**c**,**d**), and *M11* (**e**,**f**) genes analyzed by real time PCR. Relative quantification was obtained through the 2^ΔΔ*C*t^ method using actin as the reference gene. As a reference sample the untreated (control) plants from the susceptible biotype, was chosen (Cs). Data represent the average from three biological replicates and the error bars indicate the standard deviation. Leaves were collected from plants grown at 8 °C. Explanation of samples abbreviations was given on Figure legend 2. Relative expression ratios were estimated one and four days after glyphosate treatment (one DAT—diagrams on the left, and four DAT—diagrams on the right).

Interestingly, the mode of induction of *EPSPS* gene was not altered dramatically. The differences of the induction of *EPSPS* between the GS and the GR population were smaller but still statistically significant for the 1× dose (Figure 4a). Relative expression ratio declined at 4 DAT for all samples, at both light and dark conditions (Figure 4b).

On the other hand, a complete inversion of the results was detected at 8 °C for the *ABC transporters*. A two- to four-fold induction of *M10* and *M11* genes was monitored on the GS-population *versus* the GR-population. More obvious differences were detected at 4× and 8× doses (Figure 4c,e). A very interesting observation was the fact that under dark conditions the induction of *ABC-transporters* was even higher than in light conditions. Again, augmentation of relative expression ratio was observed in the GS-population at 4× and 8× doses. At four DAT, relative expression of genes declined with very few exceptions (Figure 4d,f).

## 3. Discussion

Glyphosate, being a non-selective systemic herbicide, requires full and active growth of treated plants in order to show its highest efficacy. The effects of environmental factors on glyphosate performance on targeted plants have been documented in earlier studies mainly regarding changes in its uptake and translocation [22,23,27]. The inhibition of the shikimate pathway, located in chloroplasts, has long been validated as glyphosate’s mode of action [28].

Measuring shikimate levels has long been proposed as a discrimination test between the GR and GS plants; either as an *in vivo* test [8,29] or as a leaf disk test [30]. However, clear cut differences in shikimate accumulation between GR and GS plants were not always identified, possibly due to the growing conditions, the amount of glyphosate applied and the plant growth rates [20,30,31,32]. Optimum temperature and light is considered to have a positive effect on the shikimate pathway, thus shikimate accumulation was more evident in plants maintained under light conditions after glyphosate treatment [28,33]. In our study, under dark conditions, shikimate accumulation was lower than in light conditions, presumably due to less flux in the shikimate pathway either directly and/or less efficient photosynthesis (Figure 1). Increased temperature (35 °C) and dark conditions resulted in significant shikimate accumulation on either GS- and GR-plants (Figure 1b), emphasizing the predominant role of temperature compared to light; this result is in agreement with previous reports [24,33].

As previously mentioned, the usefulness of the shikimate test has been frequently undermined by false-positive and false-negative results [8] stressing the need for standardization. In our study, it was shown that higher glyphosate load (4×, 8×) could minimize differences in shikimate accumulation between GR and GS population at either low (8 °C) and normal (24 °C) temperature conditions (Figure 1a,c). On the contrary, it was clearly shown that the most discrete differences (between GR and GS population) in shikimate accumulation were measured at 35 °C and light conditions regardless of the glyphosate dose applied (Figure 1b). At those conditions, it is expected to have the maximum flux in the shikimate pathway and, therefore, the confounding factor of glyphosate load is eliminated and the maximum differences were recorded between GR and GS population. For this reason, the above conditions are proposed as the standard ones for conducting shikimate analysis as a discriminating biochemical test to detect glyphosate resistant in *C. canadensis* plants.

Gene expression analysis was performed on GR and GS biotypes for *EPSPS*, *M10* and *M11* genes. Under light conditions at normal (24 °C) and high (35 °C) temperatures, the following points could be made:

(a) The *EPSPS* gene was significantly induced at normal glyphosate doses in the GR biotype, whereas at 35 °C no significant differences was observed between the two biotypes, suggesting that the synchronization theory of *EPSPS* and *ABC-transporter* gene expression as a glyphosate resistance mechanism is applicable only at normal temperatures;

(b) At an early stage (one DAT), across most glyphosate rates, the GR plants had a higher expression rate for both *M10* and *M11* genes compared to the GS plants (Figure 2e,f and Figure 3b,c). This result further supports previous reports about the possible role of the *ABC-transporter* genes in glyphosate resistance [30,34];

(c) GR plants had constantly higher overexpression of the above key genes only at an early stage (one DAT), regardless of the temperature. Therefore, the early time of initiation of overexpression is critical for the resistant mechanism itself, since this early overexpression (immediately after glyphosate application) of the genes secures glyphosate inactivation due to vacuolar sequestration. This result is in agreement with our previous findings [20]. Moreover, the highest *M10* and *M11* gene expression of the (1×) GS plants at four DAT (Figure 2d,f) indicates that it might be too late (at such a late stage) for the *ABC-transporters* overexpression to offer resistance protection. Also, this finding is in agreement with our previous findings [20].

Regarding gene expression at low temperature (8 °C), if GR horseweed plants are made sensitive then they should behave (biochemically and molecularly) like GS plants. In accordance to this hypothesis, two important facts were pointed out:

(a) Documented GR plants could be indeed “sensitized” (become S-GR plants) when subjected to cold and light conditions;

(b) The process of such “sensitization” is clearly correlated to the mechanism of glyphosate resistance in *C. canadensis*.

In our study, the sensitized S-GR plants were correlated with the inversion of *M10* and *M11* gene expression, but not that of *EPSPS* (Figure 4a,c,e). This finding is in agreement with previous reports about the fate of glyphosate in such plants: when horseweed GR biotypes were in cold conditions (similar to our own ones), less glyphosate was sequestered to vacuoles suggesting a role of putative *ABC-transporter* genes to glyphosate resistance [24]. Our study clearly shows, for the first time in the literature, that the lack of resistance observed at low temperatures could be attributed to low levels of expression of *M10* and *M11 ABC-transporters.*

Conclusively, the aforementioned *ABC-transporters* (*M10* and *M11*) could be key players in the *C. canadensis* glyphosate resistance mechanism due to vacuolar sequestration.

## 4. Experimental Section

### 4.1. Plant Material

Two *Conyza canadensis* populations were used in this study, one GR-population (LA7, from a citrus orchard in Lakonia, Greece), and one GS-population (AT5, from an organic vineyard with no history of glyphosate application in Attiki, Greece). The two populations were initially characterized (dose-response experiments), and seeds from the surviving plants were collected [24]. Seeds from both populations were germinated in plastic trays (350 × 295 × 50 mm) containing common peat (Klasmann TS-1, pH 5.5). Seedlings (3–4 leaves) were transplanted to pots (diameter 10 cm) and were grown in a glasshouse at 25/15 °C day/night temperature (spring time) under natural sunlight, and were well watered until reaching the required growth stage for herbicide treatment.

### 4.2. Herbicide and Temperature Treatments

Plants at the small rosette stage (8–10 leaves) were treated with glyphosate (Roundup 36 SL, 360 g·a.i./L concentration) utilizing an automated spraying chamber delivering 360 L·ha^−1^ at a pressure of 200 kPa. For dose-response experiments, the following doses were used: control, 1/2×, 1×, 2×, 4× and 8×, whereas 1× recommended dose = 533 g·a.e.·ha^−1^ and 8× = 4264 g·a.e.·ha^−1^. A randomised complete block design was used with 4 replications (each pot = replication, containing a single plant per pot), for each treatment.

In order to study the effect of environmental conditions on horseweed plants, pots were transferred into growing cabinets set at the desired conditions (160 μmoles·m^−2^·s^−1^ for a 12:12 h photoperiod) 4 days in advance to acclimatize the plants.

Regarding the temperature, the treated plants were maintained under three different conditions such as 8, 25 and 35 °C. Half of the treated plants were placed inside a wooden frame covered with black polyethylene bag to deliver the dark conditions. All the plants were observed weekly for toxicity symptoms, and special care was given to minimize the time that plants grown under dark conditions would receive light. Plant growth was recorded 28 DAT (days after treatment). For estimation of dry weight, above soil part of the plant was collected and dried in a drying chamber (90 °C, 48 h). The GR_50_ (herbicide rate causing 50% reduction on growth) was estimated using non-linear regression analysis.

### 4.3. Shikimate Measurement

For shikimate analysis, leaf sections (0.1 g) were taken from plants that were treated with glyphosate (1× and 8×), at 1 DAT and 4 DAT. The old and the very young leaves were excluded. Leaf samples were kept in a deep freezer (−80 °C) until further processed.

The determination of shikimic acid in *Conyza* leaves from GR- and GS-populations followed the procedure described by Shaner *et al.* [33] with some modifications. At 1 and 4 DAT, always in the morning, leaves of younger and fully developed leaves (5 in total) were randomly collected. The extraction of shikimic acid was performed in replicates of 100 mg of dry matter, mixed with 1 N hydrochloric acid. Leaves were incubated in the extraction solution for 24 h. Following that, a solution of 1:1 (*v*/*v*) 0.5% (*w*/*v*) periodic acid and 0.5% (*w*/*v*) sodium metaperiodate was added and samples were incubated for 3 h at room temperature. At the end of the oxidation, formation of chromophore and stabilization was achieved by adding 1 N sodium hydroxide and 0.0056 M sodium sulfite to the samples. Shikimate is determined by measurements of optical density at 382 nm using a spectrophotometer. A shikimate standard curve was developed by adding known amounts of shikimate to vials containing no leaves. Shikimate levels are presented as μg of 1 M shikimic acid per ml of HCl solution.

### 4.4. RNA Isolation, cDNA Synthesis and qRT-PCRexperiments

RNA was isolated from leaves collected at 1 DAT and 4 DAT respectively, using TriReagent (Sigma-Aldrich, Dorset, UK) according to the manufacturer’s protocol. cDNA was synthesized by preparing the following mixture in a microtube: 0.5 g of total RNA, 1 μL of dNTP mixture (10 mM each), 1 μL of oligo dt primer (100 pmol final concentration), and RNase free dH_2_O up to 10 μL. The mixture was heated at 65 °C for 5 min and cooled immediately on ice. Subsequently, 200 U of Primescript RT enzyme were added and the reaction was completed according to the manufacturer’s protocol (Takara Clontech, Mountain View, CA, USA). The cDNAs were diluted to 100 μL with sterile water, of which 1 μL was used per real-time PCR sample. The sequences of the primer pairs used to amplify *ABC-transporter* genes *M10*, *M11*, *EPSPS1* as well as *actin* gene (a housekeeping gene that was used as a non-regulated reference gene for Real Time PCR experiments) were given at our previous paper [20]. Quantitative expression analysis was performed as previously reported [20].

### 4.5. Statistical Analysis

Analysis of variance (ANOVA) was conducted for all data and differences between means were separated using Fisher’s Protected LSD test at *p* < 0.05. All statistical analyses were conducted using the Statistica 9 software package (StatSoft, Inc., Tulsa, OK, USA).
